# Examining BRCA Previvors’ Social Media Content Creation as a Form of Self and Community Care: Qualitative Interview Study

**DOI:** 10.2196/67794

**Published:** 2025-03-03

**Authors:** Mariah L Wellman, Camilla M Owens, Avery E Holton, Kimberly A Kaphingst

**Affiliations:** 1 Advertising & Public Relations College of Communication Arts and Sciences Michigan State University East Lansing United States; 2 Department of Communication University of Utah Salt Lake City, UT United States; 3 Department of Communication Huntsman Cancer Institute University of Utah Salt Lake City, UT United States

**Keywords:** BRCA, breast cancer, genetic testing, social media, breast cancer gene, content creation, self care, community care, qualitative interview, qualitative, interview, previvors, cancer previvors, genetic mutations, online, content, interviews, thematic analysis

## Abstract

**Background:**

Genetic testing has become a common way of identifying a woman’s risk of developing hereditary breast and ovarian cancer; however, not all medical providers have the necessary information to support patients interested in genetic testing, nor do they always have the proper information for patients once they have been diagnosed. Therefore, many “previvors”—the name given to those who have tested positive for the *BRCA* genetic mutation—have taken to social media to inform others about the importance of genetic testing and explain to them how to understand their test results. Historically, those desiring to speak about their medical issues online have sought out structured support groups or chat rooms; however, many previvors today are instead posting on their own personal social media accounts and creating more niche communities.

**Objective:**

This study aimed to examine why *BRCA* previvors are sharing content on their personal social media accounts and how posting online in this way serves a purpose for their larger community.

**Methods:**

A total of 16 semistructured interviews were conducted with individuals who posted about their experience being diagnosed with the *BRCA* genetic mutation and their subsequent treatment on their personal social media accounts, specifically for followers interested in their medical journey. The interviews were recorded, transcribed, and coded by an experienced qualitative researcher and a graduate student using inductive techniques, and a reflexive thematic analysis was applied to the transcripts.

**Results:**

The results suggest *BRCA* previvors want to control the narrative around their personalized medical experiences rather than participating in existing groups or chat rooms. Controlling their own story, rather than adding to existing narratives, gives previvors a sense of control. It also allows them to set boundaries around the types of experiences they have online when sharing their medical journey. Finally, previvors said they feel they are serving the larger *BRCA* community by each sharing their individual journeys, to hopefully avoid stereotyping and homogenizing the experience of patients with *BRCA* genetic mutations.

**Conclusions:**

Research with the objective of understanding the experiences of *BRCA* previvors should include exploring how and why they talk about their journeys, especially due to the lack of knowledge *BRCA* previvors say many of their medical providers have. We suggest further research should examine how other patients with the *BRCA* genetic mutation, especially racial and ethnic minority patients, are navigating their own content creation, especially considering content moderation policies that social media platforms are continuing to implement that directly impact users’ ability to share about their medical experiences.

## Introduction

As the world’s most commonly diagnosed cancer, the urgency to address breast cancer and support those with an increased risk of developing this disease has become even more important [1]. In 2020, the World Health Organization reported 2.3 million globally diagnosed cases of breast cancer, along with 685,000 deaths attributed to the disease [1]. In many cases, breast cancer occurs sporadically. However, genetic factors do play an influential role in 10% to 15% of all cases [2]. Like breast cancer, the risk of ovarian cancer also increases in women with age, along with having inherited genetic traits such as a *BRCA1* or *BRCA2* gene mutation [3]. In 2020, ovarian cancer was the third most prevalent and lethal gynecological cancer worldwide [4-6].

With continued research and treatment of breast and ovarian cancer, it has become more common for medical providers to encourage genetic testing, especially for those whose relatives who have been diagnosed with cancer [7-9]. Women who test positive for a *BRCA* genetic mutation are at a higher risk of developing hereditary breast and ovarian cancer, which impacts their health, reproductive choices, and identity [10,11]. Within their lifetime, individuals with a *BRCA* mutation have up to a 75% increased risk in developing breast or ovarian cancer [12]. Those who do test positive for a *BRCA* genetic mutation and have not yet been diagnosed with cancer are known as “previvors,” a distinction for those at higher risk for developing cancer [10].

Due to the aggressive nature and reduced life expectancy associated with breast and ovarian cancer, it is necessary that the public, especially those who have a family history of breast and ovarian cancer and those who have already tested positive for the genetic mutation, are informed about the risks associated with the mutation that they carry and its impact on one’s health and family planning [7]. However, previously published research suggests not all medical providers have the information previvors are looking for [7]. Previvors are looking for medical information and resources from various in-person and online sources regarding the options available to them should they desire to undergo surgery and reconstruction, just undergo surgery, or simply monitor themselves over time [11]. However, they are also looking for emotional support from loved ones, especially those who also have this genetic mutation and can provide advice and social support [7,11]. Social media and the ability to create content is a convenient and valuable tool for such purposes, and thus, researchers are exploring how social media is used by those with a *BRCA* genetic mutation [7,11,13].

*BRCA* previvors, cancer survivors, and those living with cancer use social media, online blogs, and internet chat rooms to connect with others [7,11,14,15]; however, much of the research on these communities is done through the lens of those seeking information, support, and connection rather than creating it [16,17]. When patients first began searching for information and for others in similar situations, they often did so through online platforms that afford a built-in community like chat rooms or message boards [15]. More recently, however, patients are using their own personal social media accounts to tell their stories on an individual level, hoping to build community on their own terms [7,11,13]. Individuals with breast and ovarian cancer communicate information that is personal or social on their individual accounts, with two-thirds of posts conveying actual experiences or providing support to others [18]. Using social media in this way distracts users from the stresses they may be experiencing that are caused by new, recurring, or terminal illnesses [19].

As a shift has occurred from group-centered platforms to individual storytelling spaces, there remain significantly fewer social media posts engaging with *BRCA* and genetic testing [11]. However, there are social media users increasing the amount of *BRCA* content being created, and health communication and internet scholars must pay attention to these individuals and their content [7,11]. As mentioned earlier, most research on social media and *BRCA* previvors has examined how previvors find information, rather than create it themselves, although the lens through which scholars are examining this content is shifting [7,11]. Considering the limited information available, this study aims to add qualitative insights regarding the creation of content by and for *BRCA* previvors. To accomplish this, we conducted semistructured interviews with *BRCA* previvors who create social media content related to their health condition and identity as previvors. Trends and themes were analyzed across the interview transcripts regarding *BRCA* previvors, social media, content creation, and connection within digital spaces. At the start of this study, we asked the following research questions: (1) Why do *BRCA* previvors create content on their personal social media accounts rather than in the digital communities created for *BRCA* previvors such as Facebook (Meta) groups? and (2) How do *BRCA* previvors perceive their social media content creation serving a purpose to their larger community?

Through qualitative interviews with a selective group of *BRCA* previvors who post personal medical experiences on their own social media accounts, this study offers a novel perspective on the trend of sharing health information online. Through prioritizing the words of our participants, we believe this study provides insight into the thoughts, feelings, and experiences of individual *BRCA* previvors and can help researchers and health care providers understand the ongoing needs of patients with *BRCA* genetic mutations.

## Methods

### Recruitment and Data Collection

To examine *BRCA* previvors’ experiences creating social media content for themselves and for their larger community, we used a qualitative interview approach. We prioritized recruiting individuals living in the United States who have been found to carry a *BRCA1* or *BRCA2* genetic mutation, resulting in an increased risk for developing breast or ovarian cancer, and publicly shared about their medical experiences on social media, specifically Instagram (Meta). First, the primary investigator conducted a purposive sample by searching for *BRCA* previvors active on Instagram through hashtags including “#brca,” “#brca1,” “#previvor,” “#brcagene,” and “#breastcancerprevivor.” Once the accounts were populated, the investigator reached out to potential participants who listed their email addresses publicly on their Instagram accounts. We acknowledge that the algorithm on social media platforms like Instagram may have played a role in our sample procedure; however, we were interested in interviewing individuals who acted as influencers, opinion leaders, and public figures in *BRCA* online communities and, therefore, recognized that the most popular accounts may be the most accessible by using this purposive sampling method. Upon finding these individual accounts with publicly listed email addresses, the primary researcher sent an email detailing the study objectives and requesting potential participants to respond if interested. Of the total study participants (N=16), 15 identified as women and 1 identified as nonbinary. The nonbinary participant noted that they are often perceived as female and check “woman” as the gender on their medical paperwork for insurance purposes. All participants were White presenting; however, 2 identified as Hispanic and 2 identified as Ashkenazi Jewish. The latter group noted their ethnicity as important to their *BRCA* status, as 1 in 40 Ashkenazi Jewish women have a *BRCA* genetic mutation [20]. Participants were all over the age of 18 years at the time of the interview, and all confirmed that they are actively posting about their *BRCA* experiences publicly on one or more social media platforms.

Participants who met all eligibility criteria were then scheduled for a virtual, semistructured interview over Zoom (Zoom Video Communications). Consent forms were signed before the interview was scheduled, and verbal consent was confirmed once again before the interview began. All interviews were conducted by the primary investigator, and each participant was provided the opportunity to turn their camera off before the interview began to further protect their identity. The interviews included questions related to the participants’ experiences with *BRCA*, their desire to create content on their personal social media accounts, their interactions with the *BRCA* community, their understanding of their previvor identity, and their relationship with health care providers. Each interview lasted between 45 and 60 minutes. The interviews were audio recorded for accuracy, and upon completion of the discussion, each interview audio file was labeled with a participant number. After all personal identifying information was removed from the files and the audio recordings, they were transcribed by a professional transcription service.

### Data Analysis

Reflexive thematic analysis [21,22] was applied by the principal investigator and supported by a graduate research assistant. This analysis method has been applied in recent studies of women with *BRCA* genetic mutations and how they share social media content, build community, and understand their identity as *BRCA* previvors [7,10,11]. First, the principal investigator and graduate research assistant split up the 16 transcripts and manually coded 8 each. They then met and discussed overlapping patterns in the data related to why *BRCA* previvors post on social media about their previvor experiences and the role content creation plays in the act of community building and caring for oneself through various *BRCA* experiences. After the first meeting, the principal investigator and graduate research assistant switched transcripts and coded the other set of 8 transcripts. They then met a second time to finalize the themes present that related to the initial research questions. The team elected to follow this 2-stage coding process recommended by previous researchers to maintain the rigor of the method. All direct quotes provided in the results section below are anonymized and referred to by participant number.

### Ethical Considerations

As this study involved human participants, the University of Utah’s institutional review board approved the study protocol (IRB_00144720) before we began. An informed consent document was sent to each participant for review before they elected to participate. The document described the nature of the study, the types of questions asked, the protocol researchers followed to protect participant anonymity, and information regarding their rights as research participants including the opportunity to decline to answer any question and stop the interview at any time. The primary researcher kept a password-protected document on an external hard drive, which included the demographic information of each participant, including their name and participant number, to protect their identity but provided a reference sheet for use when needed. All personal identifying information was removed from the files and the audio recordings. The participants who completed the interview process were compensated in the form of a US $50 gift card.

## Results

### Posting on Social Media Fills a Gap in the Narrative of BRCA Previvorship

The *BRCA* previvors we interviewed said they create content on their personal social media accounts rather than in digital communities, such as Facebook groups, because they like to be able to control the narrative around their own personal journey and fill a gap in society’s understanding of what it means to be someone living with a *BRCA* genetic mutation. *BRCA* previvors desire connection beyond existing previvor and survivor communities, and many noted they create content because they want to share every step in their journey with others, regardless of whether those watching have *BRCA* genetic mutations or not. One previvor explained that in her opinion, nothing is off limits when you share about *BRCA* on your own personal page.

I’ve shared every step of the way and there’s nothing, in my opinion, that I shouldn’t share.

P11

Similarly, many of the previvors interviewed have shared in-depth videos and photos of surgery scars, drain removals, breast reconstruction, nipple tattoos, and more.

Sharing whatever they choose, whenever they choose results in previvors feeling a deeper sense of autonomy over their lives and control over when and how they talk about *BRCA* genetic mutations. They do not want to talk strictly about the clinical aspects of their genetic mutation; they want to talk about sex, desire, hormones, body image, and how testing positive for *BRCA* genetic mutations impacts their identity as a wife, daughter, sister, mother, and friend. One previvor explained that she wanted other women with *BRCA* genetic mutations who follow her to understand that they may change, both inside and out, but they can still be the same woman they were before being diagnosed and they do not have to allow their *BRCA* genetic mutation to take away who they once were.

I took people on my journey to show them that you can be strong enough to go through it and come out on top of it and no matter what you look like, it's still you.

P14

When discussing *BRCA* genetic mutations from the clinical perspective, previvors say they sometimes feel like their condition is overgeneralized, which results in feelings of homogeneity, especially in the eyes of their health care providers. In previous studies, previvors noted some providers make assumptions about the care they desire and do not always leave space for individuals to request a treatment plan that works best for them [7]. One previvor shared her experience defending her decision to forego reconstructive surgery in favor of a flat chest, something she did not know was possible from only speaking to her providers. She found other options through social media and now posts on her Instagram account to help others.

I hope people see (my chest) and they’re like ‘Oh, we can go so many different directions for our BRCA decisions. We don’t just have to have reconstruction or like just a flat chest like’—there’s things where you can make it feel more like you or learning how to make your body feel like yours.

P15

Showing a personalized perspective of the previvor experience on social media, previvors say, offers others a chance to see what daily living with *BRCA* genetic mutations is like, especially for those diagnosed at a young age. Previvors interviewed noted that many young patients with *BRCA* genetic mutations have different goals regarding their lifestyle, and Facebook groups and online chat rooms do not reflect those previvors and their understanding of *BRCA* genetic mutations. One previvor noted:

Facebook is just a different type of platform; people are posting pictures or questions or lamenting some situation. Whereas Instagram, it’s more of a story of my life and not just a way to complain. So, I do think it was more helpful to be able to control the content on Instagram and not have anybody disqualify my experience. I think that was my biggest irritation with the Facebook groups. I felt like I was being questioned for whatever decision I was leaning towards. And this way I could just be like, ‘This is what I’m doing. These are my risks. Just wanted you to know.’ Not open for comment or questions or your opinions.’

P17

Younger previvors have turned to Instagram and TikTok (ByteDance) to find others with *BRCA* genetic mutations who have similar experiences and opinions regarding living with a genetic mutation and seeking possible treatments. For example, many younger previvors do not want to take the surgical route immediately after being diagnosed, as they are concerned about infertility, losing the opportunity to breastfeed a future child, and how their future may be dictated by decisions they are making from a young age. One previvor shared that she began posting because she felt like nobody was discussing *BRCA* genetic mutations from the perspective of a younger woman, especially a woman who wanted to have kids one day.

I started relying on social media to not only like see what other people were saying, especially people my age, but then being able to start talking about it from my personal perspective because I know my friends especially before 30 are like not thinking about (having kids) yet.

P18

For this previvor, posting on her social media accounts specifically on what young previvors need to be thinking about is important because she may be able to help another young patient with *BRCA* genetic mutations make a decision that was not offered by their health care provider.

### Previvors View Content Creation as an Act of Self and Community Care

For *BRCA* previvors, creating content on their own social media accounts focused on their experiences helps them build a sense of autonomy over having a *BRCA* genetic mutation while taking care of themselves and offering support to others with a genetic mutation. This act of self-care and care for the community was a common explanation for why our interviewees started posting on their personal social media accounts. As one previvor said:

I wanted women to see that they’re not the only ones going through it, that there are other women out there and that it will be okay and that you’re beautiful even with all the scars and that you’re still a woman.

P14

Many previvors said their understanding of themselves as a woman shifted once they were diagnosed, and they believe this shift is common among patients with *BRCA* genetic mutations. *BRCA* previvors expressed feeling afraid when initially diagnosed and unsure of what the future would hold for them as women, mothers, wives, daughters, and friends. Posting on social media allowed them to discuss their understanding of womanhood after diagnosis, which became an act of self-care and a way to give back to others in their community. Later in the interview, the same previvor shared how she felt upon being diagnosed and how her social media content creation helped her feel more like herself.

For a long time, you don’t know who you are anymore. BRCA is—you were dealt this card, and you try to deal with it, but everything that makes you a woman is taken away from you. You don’t know—it’s like I know my body or my breasts or my ovaries don’t define me as a person or a woman, but that is what makes us a woman and when that’s taken away from you, you have to kind of learn to love yourself again in a different way.

P14

Posting on social media provided an outlet for previvors while they learned to love their new bodies, minds, and spirits after diagnosis. In the interviews, some participants said they felt like their *BRCA* genetic mutation initially stole their voice and that they no longer had a say over their own lives. However, sharing on social media gave them the opportunity to speak about whatever they were going through whenever they felt like they needed to. For one previvor, the posting was a form of therapy.

For me, it was almost like a journal, kind of therapeutic to put it out there in the world and be very open about it and then have—I was surprised how many of my friends and people that follow me that have had breast cancer, how supportive they were. I really expected them to be like the nursing staff, like ‘Oh, you don’t have real cancer,’ but they were all super supportive.

P03

The therapeutic nature of posting on individual social media accounts was more helpful for these creators than sharing in chat rooms or Facebook groups, many said. Previvors felt like some chat rooms and online groups have become spaces where toxic positivity runs rampant but, at the same time, can be a place where previvors are judged by others regarding their care and treatment decisions. In the interviews, some previvors described the breast cancer survivor community, and more recently, the previvor community, as a space where media and large organizations “pink-wash” the disease and, thus, take away from the authentic lived experiences of breast cancer survivors and previvors [23]. These actions, while they can result in financial gain for some, do not provide the kind of support many previvors say they are looking for. Another previvor explained that the lack of authentic experiences publicized through media outlets and large organizations does a disservice to all people.

There was just a lack of representation and of candid, honest representation. I wanted someone that I could relate to. It wasn’t just like this sob story because like parts of it were funny and weird and so full of love. I know that my story is not everyone else’s, but like somebody’s going to be able to relate to that, right? And also…(I wanted to) talk about sex and like all these things that I’m so used to talking about openly, but other people don’t. So, I was like ‘All right, I’ll just write it all. Hopefully, it will help somebody.’

Others agreed, saying:

it’s so amazing to have the chance to share your voice, no matter what it be about,” and “I post the ugly. I post the good and the bad. I want people to know there is the ugly part of it, but there can be a good part to it too.

P16

Ultimately, previvors agreed that sharing on their personal social media accounts is a form of community care and a way to continue building a supportive community. As one previvor noted:

I can take the fear that some women have, that I can show them that ‘You can do it. You will come out on top of this. Don’t let the BRCA run your life. You run BRCA. You can do something’

P04

Previvors on social media want others to know they are not alone and that there are spaces where they can be exactly who they are without judgment, even if they must create it themselves. As one previvor summarized:

I think if we keep (our journeys) to ourselves, then we’re not doing any good

P14

## Discussion

### Principal Findings

Studies describing the content creation of *BRCA* previvors are a relatively understudied area of health communication and medical internet research. The previvors we interviewed noted that they post on their personal social media accounts rather than online spaces created specifically for group communication like chat rooms and Facebook groups because it allows them to control the narrative around their *BRCA* experience. This finding provides greater nuance to research that claims when patients interact online with others, they feel a sense of social support [7,11]. In addition, this finding provides a greater understanding of those individuals who not only seek health information online [16,17] but also create it themselves.

For previvors, controlling the narrative allowed them to speak on the topics they deemed most important and gave them the power to remove or block those who judged or critiqued their choices. This gave our participants a greater sense of power over their situation and reduced their fears around the possibility of pushback from other previvors. Some said they received negative responses from others with *BRCA* genetic mutations in spaces like Facebook groups but did not receive that feedback on their own social media accounts. *BRCA* previvors also believed that posting on their personal social media accounts served a greater purpose beyond themselves. They envisioned their content to serve the larger *BRCA* community in their quest to take care of each other, provide guidance regarding treatment plans and surgery options, and increase the visibility of *BRCA* genetic mutations itself. This finding extends previous studies that posit the discourse within online spaces has the potential to extend offline and impact the care patients receive [7,11,13,16,17].

While our study participants did not bring up any potential risks or negative consequences to sharing their personal health stories online, it is important to consider privacy concerns when one shares personal health information on social media platforms [24,25]. In addition, there is potential for misinformation to spread in these online health spaces, especially as patients continue to share their experiences on individual accounts that are more difficult to monitor [26,27]. While our participants did not mention misinformation as being common within *BRCA* spaces, as the communities grow, the issue will be important for researchers to consider when evaluating the benefits and risks of these individual storytelling spaces.

### Conclusion

This study was strengthened by the in-depth interviews given by each participant, but the study was limited by the number of interviews conducted and the interview process itself. Due to COVID-19 limitations, all interviews were conducted through videoconference, often reducing the opportunity for the interviewer to analyze participants’ body language. In addition, the reliance on virtual interviews means that while we were able to communicate with previvors across the country, each participant’s experiences with their local health care system may differ [28]. In addition, this study relied on the most popular *BRCA* social media accounts to recruit participants. On platforms like TikTok and Instagram, the algorithm may dictate whose content is promoted and whose is not, which results in a skewed sample, but this does not necessarily mean these stories are less worthy of being told. However, the algorithmic bias present on social media platforms can often result in a list of potential participants who are neither racially nor ethnically diverse. Our study skewed primarily toward White women, which mirrors results found in other recent studies of *BRCA* previvors on social media [7,11]. We recognize the lack of scholarship exploring racial and ethnic minority patients who have been diagnosed with the *BRCA* genetic mutation, especially analysis of their online presence and their desire to create digital spaces for the needs of their specific community, and we urge scholars to continue prioritizing these individuals.

Future research should also consider the practice of moderating and censoring of medical conditions based on platform policies, as some of our participants noted their concerns around content being removed or accounts being closed altogether because platforms may deem their content regarding reconstruction and recovery as sexual in nature. Social media platforms are evolving, and the results of their impact on health behaviors and outcomes remain mixed. Therefore, we believe researchers should continue to examine the therapeutic and supportive role of personal social media content across acute and chronic health conditions [29]. In addition, social media literacy continues to be key in improving health decisions, and research suggests platforms themselves can help in this process [30], especially as more patients share about their health conditions on their personal accounts. As more *BRCA* previvors take to social media platforms to share their stories and experiences with genetic testing, treatment, patient-provider interactions, and more, researchers should consider conducting studies that rely on quantitative, qualitative, and critical rhetorical methodologies to provide a larger body of work in this specific subfield.

## Appendix

Multimedia Appendix 1 Standards for Reporting Qualitative Research (SRQR) Checklist.

## Data Availability

The datasets generated or analyzed during this study are available from the corresponding author on reasonable request.
