# Supplementary figures and images for: Examining BRCA Previvors’ Social Media Content Creation as a Form of Self and Community Care: Qualitative Interview Study
Source: J Med Internet Res. 2025 Mar 3;27:e67794. doi: 10.2196/67794 (PMC11914846; doi:10.2196/67794)

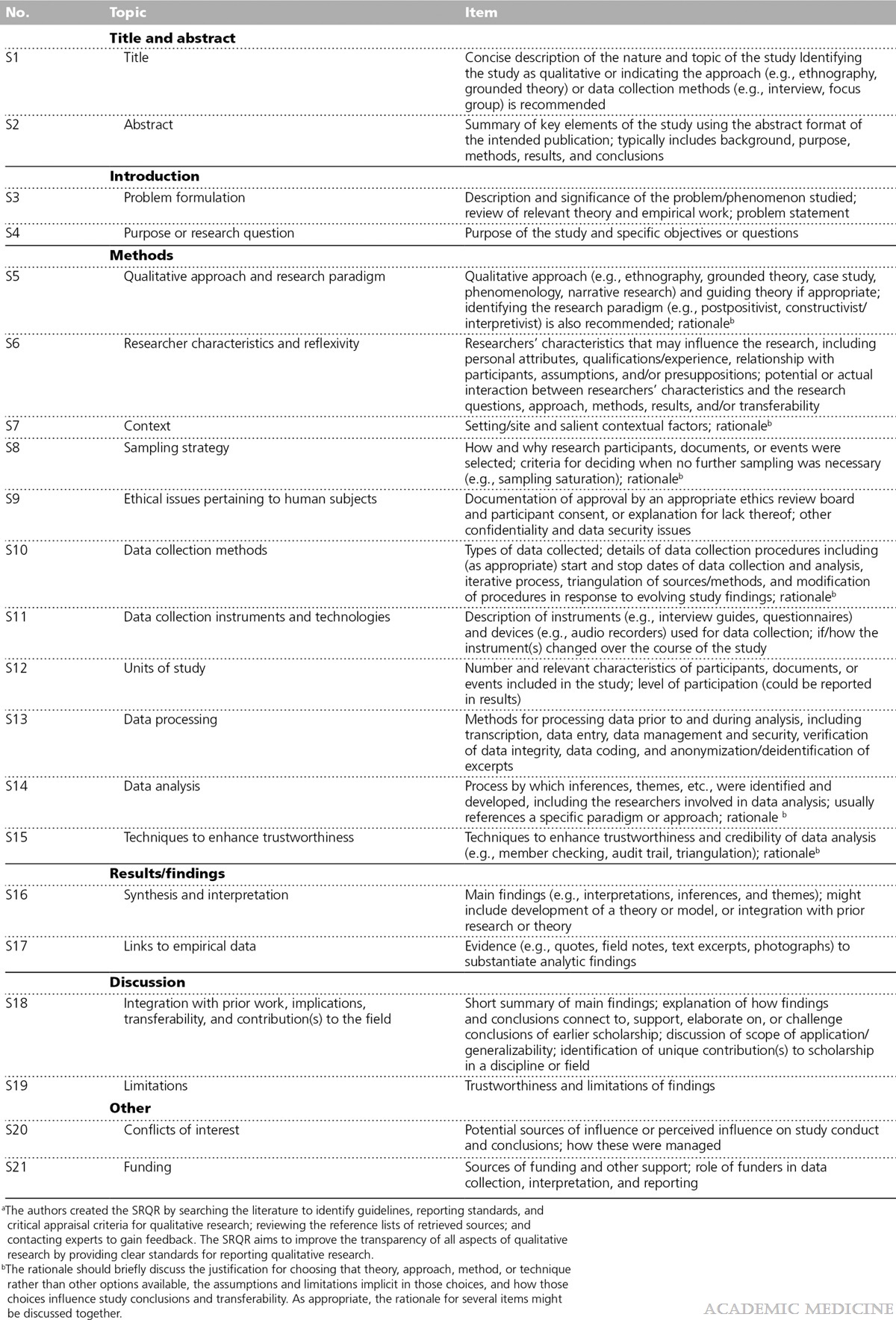

Supplement: Multimedia Appendix 1 [file jmir_v27i1e67794_app1.docx]
